# Influenza Vaccination of Healthcare Workers Is an Important Approach for Reducing Transmission of Influenza from Staff to Vulnerable Patients

**DOI:** 10.1371/journal.pone.0169023

**Published:** 2017-01-27

**Authors:** Andrew C. Hayward

**Affiliations:** Institute of Epidemiology and Health Care, University College London, London, England; University of Chieti, ITALY

Influenza vaccine is effective at preventing influenza in healthy young adults.[1] The strategy to encourage influenza vaccination for health care workers is partly based on the simple notion that this can reduce the risk of staff acquiring and transmitting influenza to vulnerable patients and thereby reduce associated morbidity and mortality. A number of investigators have attempted to demonstrate the impact of influenza vaccine in healthcare workers on patient outcomes.[2–5] They have used the most robust methodology available to them for assessing group interventions, namely the cluster randomized controlled trial. These trials have focused on long-term care facilities (LTCF) for the elderly. Such facilities house frail elderly patients for months or years in congregate settings. Outbreaks of respiratory tract infections in LTCF are extremely common. For example a surveillance study in Toronto identified 46 outbreaks across 5 homes in 3 years (more than three outbreaks per home per year with an average case fatality rate of 8%).[6] Despite high levels of resident vaccination, low vaccine efficacy in the elderly means that they remain vulnerable to influenza and its complications. Residents have very high rates of mortality and hospitalization especially during periods of influenza circulation. Even when residents have regular visitors, their main contact with the outside world is via healthcare staff. Long-term care facilities, therefore, provide an opportunity to demonstrate proof of the principle that healthcare worker influenza vaccination can prevent influenza and associated morbidity and mortality in their patients. Cluster randomized trials of staff vaccination in these settings have consistently shown benefit to residents (2–5) and this evidence has been used to support influenza vaccination of healthcare workers more generally. Since part of the rationale for vaccinating healthcare workers is patient protection some employers have chosen to make staff vaccination a condition of employment, which has led to legal challenge. De Serres and his co-authors present a number of arguments based on their assessment of the evidence as to why they think mandatory vaccination is not warranted.[7]

In our study [4] we aimed to test the hypothesis that a campaign promoting influenza vaccination of staff would reduce influenza-related morbidity and mortality in elderly residents of long-term care facilities. The study was based in a national chain of LTCF providing 24 hour nursing care (i.e. the subset of LTCF catering for the most frail elderly). We hypothesized that the effect of the vaccine would be confined to periods when influenza was circulating and undertook the study over two years to minimize the risk of being unable to demonstrate an effect in a year with low levels of influenza circulation. The study took place over two winter seasons, the first of which had considerably higher levels of community influenza activity and influenza related deaths than the second. Although the first year has been reported as a vaccine mismatch year, meta-analyses show that vaccine effectiveness in healthy adults is not usually significantly lowered during mismatch years [1] [8], and a study of mismatched inactivated influenza vaccine in Canadian adults during this year showed a 62% relative risk reduction in laboratory confirmed influenza.[9] During the period of more intense influenza circulation we found highly statistically significant reductions in residents’ influenza like illness (9 fewer reports per 100 residents in intervention vs. control homes– 95% CI 3–14 p = 0.004), GP consultations for influenza like illness (7 fewer consultations per 100 residents 95% CI 2–12 p = 0.002), hospitalisations with influenza like illness (2 fewer hospitalisations per 100 residents -95% confidence intervals 0–3 p = 0.009) and all cause mortality (5 fewer deaths per 100 residents in intervention compared to control homes– 95% CI 2–7, p = 0.002). Although the findings were highly significant the confidence intervals indicate a wide range of uncertainty about the scale of the benefit. We found no significant decreases in any of our outcomes during periods when influenza was not circulating in the community or in the second year when influenza rates were substantially lower than the first. In our discussion, based on our own findings and those from other studies, we concluded that healthcare worker vaccination provides an important level of resident protection in long-term care facility settings. While we claimed that the findings may be generalizable to other settings we did not intend to imply that the extent of the benefit would be similar in other settings. Indeed we think the effect is likely to be substantially greater in long-term care facilities for frail elderly residents than in the acute care setting or in long term care facilities catering for less frail patients.

De Serres assessment is based on four main points. First, De Serres et al assert that the cluster-randomized trials violate the principle of dilution whereby the greatest reductions should be observed in the most specific outcomes (e.g. greater relative reductions would be expected for laboratory confirmed influenza than for all cause mortality). Since the studies were not adequately powered to assess which outcomes had the greatest reductions and the confidence intervals for all outcomes overlap we do not agree that the studies individually or collectively violate this principle. For example in our study the reduction in influenza like illness was greater than the reduction in GP consultations for influenza like illness which was in turn greater than the reduction in hospitalisations with influenza like illness. The point estimate of the reductions in all cause mortality (5 fewer deaths per 100 residents) was greater than that for hospitalisations related to influenza like illness (2 fewer hospitalisations per 100 residents). According to De Serres et al this would violate the principal of dilution. However the confidence intervals for these measures overlap considerably (2–7 and 0–3 respectively) indicating that we do not know which measure had the greatest reduction.

Secondly De Serres et al highlight that a high proportion of averted deaths were not labelled as deaths with influenza like illness. We do not think this makes our results less plausible firstly because many deaths will have occurred in hospital but were reported by nursing home staff so they will not have been able to assess whether they had influenza like illness at the time of death. Secondly influenza may trigger a chain of events leading to death but at the time of death such patients may no longer have an influenza like illness. Since our data were collected as aggregate total event numbers for each outcome we cannot tell how many of those who died had a recent influenza like illness. Thirdly the symptomatology of influenza may be very vague in the frail elderly with multiple co-morbidities, especially as they approach death. Finally, as discussed above, the statistical uncertainty around estimates of effect mean that whilst we can be confident that there was a highly significant reduction in all cause mortality we do not know whether this represents a greater reduction than that in influenza like illness deaths. We therefore reject De Serres et al’s assertion that these are paradoxical findings or that they should “reinforce concerns about the reliability and validity of the study’s conclusions.”

Thirdly, De Serres et al highlight that in some trials protective effects were also observed outside the time period when national surveillance detected influenza activity. This was not the case for our study. We found no effect of the intervention for any of our outcomes outside the period of influenza circulation, strengthening the credibility of our results. However it should be noted that influenza surveillance only picks up the tip of the iceberg of influenza activity such that early community transmission may be missed.[10] Periods of circulation identified through national level surveillance often do not coincide with periods of circulation identified at local level.[11] Sporadic outbreaks of influenza have also been observed in LTCFs outside normal periods of influenza circulation.[12] Consequently the fact that other studies found some of the protective effects outside the period when national surveillance indicated circulation of influenza does not mean their results are implausible.

Finally, De Serres et al’s main criticism is that if the numbers needed to vaccinate (NNV) to prevent one death in our study were extrapolated to all LTCF staff in the US the number of deaths averted would be considerably greater than the annual number of deaths estimated to be due to influenza in the US. Also, if extrapolated to all hospital health care workers in the US the predicted number of deaths averted would exceed the number of influenza deaths in the US during the 1918 pandemic. Whilst we proposed that the concept of staff vaccination protecting patients was generalizable to other settings we did not claim that the estimate of the NNV would be equivalent in other settings, indeed we think it self evident that this would not be the case.

In making their extrapolations De Serres et al first appear to project the findings of our study to the whole of the US Long Term Care Sector. Our study took place in LTCFs for the elderly in homes that have 24 hour nursing care. As such they cater for the most vulnerable elderly patients with high levels of co-morbidity who are too ill to live at home or in communal establishments with less intense support. It is not appropriate to seek to generalize the NNV derived in this setting to the broader LTCF sector where residents are likely to be substantially less vulnerable to the effects of influenza and where they may have more contact with the outside world. It is also not appropriate to seek to generalize this NNV to the acute hospital setting where length of stay is short, patients are on average younger and patients have contact with many people other than health care workers. In addition, De Serres et al focus solely on results derived from the point estimate when assessing the credibility of the NNV figure whilst ignoring the 95% confidence intervals around this estimate (5.8–20.4), which indicate a high level of uncertainty about the scale of the effect size. De Serres et al also make the assumption that the NNV would not increase as vaccination rates increase (e.g. assuming that increasing staff vaccination from 50% to 100% would have the same impact as increasing vaccination rates from 0 to 50%). This is unlikely to be the case due to herd immunity effects. De Serres et al’s extrapolations to infer the impact of complete staff vaccination to settings that differ considerably from that of nursing homes for frail elderly should therefore not be used to undermine the validity of our findings in the nursing home sector.

In England and Wales on the night of the 2011 census there were 389,102 individuals usually resident in long term medical and care establishments but only 123,137 were aged 65 years or over and living in long-term care facilities with 24 hour nursing care (i.e. the population covered in our trial). [13] In our study the staff:resident ratio was 1.27 suggesting there would be 157,211 nursing home staff in England and Wales—or if extrapolated to the whole of the United Kingdom, 176,933. Our study increased overall staff vaccination rates from 5% to 35% (although 48% of full time staff were vaccinated). This would equate to 53,080 additional vaccinations nationally. Given our estimates of NNV this suggests that achieving equivalent staff vaccination levels as those obtained in our study across similar homes in the UK in a year with a similar level of influenza activity would avert 6473 deaths (95% CI 2602–9152). A recent modelling exercise estimated that on average there are around 14,000 annual cardiorespiratory deaths in the UK attributable to influenza in those aged 65 years or older.[14] These deaths are further concentrated in those aged over 75 and those with comorbidities. High annual variation in influenza activity means these estimates have very high standard deviations such that plausible annual attributable mortality figures can be several times this level. We therefore do not think that the range of estimates we give for NNV is implausible. We do not think that this scale of benefit would be realistic outside the specific setting of Long Term Care Facilities for the elderly with 24 hour nursing cover. The cluster-randomised trials of staff vaccination in long term care facilities collectively provide strong evidence of the principle that staff vaccination can protect patients but further work would be needed to establish the potential scale of the benefit in different settings.

De Serres et al attempt to “recalibrate” the NNV for the hospital sector. Using a wide range of assumptions they estimate that 6000 to 32,000 hospital workers would need to be vaccinated before a single patient death could potentially be averted. In assessing these estimates they use measures of the numbers of influenza related deaths reported in surveillance studies that are assumed to be hospital acquired and therefore potentially preventable by staff vaccination. It should be noted that without very rigorous application of swabbing and PCR testing of all those developing respiratory symptoms in hospital the number of hospital acquired cases and consequently the number of associated deaths is likely to be underestimated, and the NNV to be overestimated. In addition the numbers of potentially preventable deaths used were based on surveys in hospitals that already had staff vaccination programmes achieving staff vaccine uptake of 40–70%. The estimates therefore do not account for cases of influenza and associated deaths that may already have been prevented through this level of vaccination.

Doubtless, the debate as to whether or not staff influenza vaccination should be a condition of employment will continue. If avoiding patient death was the sole aim of staff vaccination a reasonable economic case could be made for staff vaccination in hospital settings at the NNV estimated by De Serres (influenza vaccine costs less than $2 per dose). When factoring in the prevention of unnecessary illnesses in patients and staff, associated health service costs and the avoidance of staff sickness absence, the economic case is still more compelling. Economic arguments cannot, however, indicate a level of protection at which vaccination should be made a condition of employment. Regardless of such enforcement measures health care workers need to consider their professional duty to take reasonable actions to protect their patients from infection. De Serres et al argue that more broadly protective practices, such as staying home or masking when acutely ill could be alternative approaches to protection, but unlike staff vaccination the effectiveness of these measures has not been assessed. The fact that viral shedding precedes symptom onset by around 24–48 hours, that many people with laboratory-confirmed influenza have mild symptoms that may be hard to distinguish from the common cold and that many infections are asymptomatic would minimise the effectiveness of such measures.[10,15,16] Avoiding influenza through vaccination is an important approach for healthcare workers to take to avoid unnecessarily passing infection on to their vulnerable patients.
